# Die Bedeutung der Konzepte One Health und Planetary Health für die Umweltmedizin im 21. Jahrhundert

**DOI:** 10.1007/s00103-023-03711-6

**Published:** 2023-06-02

**Authors:** Tomke Zschachlitz, Romy Kümpfel, Hildegard Niemann, Wolfgang Straff

**Affiliations:** 1 Fachgebiet II 1.5 Umweltmedizin und gesundheitliche Bewertung, Umweltbundesamt, Corrensplatz 1, 14195 Berlin, Deutschland; 2 Abteilung 2 für Epidemiologie und Gesundheitsmonitoring, FG 24 Gesundheitsberichterstattung, Robert Koch-Institut, Berlin, Deutschland

**Keywords:** Planetare Grenzen, Umwelt und Gesundheit, Klimawandel, Global Health, Public Health, Planetary boundaries, Environment and Health, Climate change, Global health, Public health

## Abstract

Im 21. Jahrhundert führt eine Anhäufung komplexer Krisen wie Klimawandel, Biodiversitätsverlust, Umweltverschmutzungen, Kriege und Pandemien zu ökonomischen, sozialen und gesundheitlichen Problemen für Menschen jetziger und zukünftiger Generationen. Diese Probleme sind im Wesentlichen auf die Missachtung natürlicher Regenerationskapazitäten von Ökosystemen zurückzuführen. Gesundheitsansätze wie One Health und Planetary Health haben seit Beginn der 2000er-Jahre an Popularität gewonnen und finden verstärkt Anwendung in der Politik, der Wissenschaft und in den Gesundheitsberufen. Auch die Umweltmedizin wird durch die wachsende Zahl von Krisen und Umweltproblemen sowie das zunehmende Interesse an den Konzepten One Health und Planetary Health beeinflusst.

In diesem Diskussionsbeitrag wird zunächst die Entwicklung der beiden Konzepte dargestellt. Danach wird auf ihre Bedeutung für die Umweltmedizin eingegangen. Ärztinnen und Ärzte, die sich mit umweltmedizinischen Themen befassen, müssen bei der Beurteilung von Umwelteinflüssen heute sowohl lokale als auch globale Ursachen und Gesundheitseffekte bedenken, was Entscheidungen komplizierter machen und zu Zielkonflikten führen kann.

## Abstract (en)

In the 21st century, an accumulation of complex crises such as climate change, biodiversity loss, environmental pollution, wars, and pandemics is leading to economic, social, and health problems for people of current and future generations. These problems are essentially due to the disregard of natural regenerative capacities of ecosystems. Health approaches such as One Health and Planetary Health have gained popularity since the early 2000s and are increasingly used in politics, science, and the health professions. Environmental medicine is also influenced by the growing number of crises and environmental problems and the increasing interest in the concepts of One Health and Planetary Health.

In this discussion paper, the development of the two concepts is first presented. Then, their significance for environmental medicine will be discussed. Health professionals working on environmental medicine issues must now consider both local and global causes and health effects when assessing environmental impacts, which can complicate decisions and lead to conflicting objectives.

## Einleitung

Im 21. Jahrhundert nehmen vom Menschen verursachte Risiken und Bedrohungen zu. Beispiele hierfür sind der Klimawandel, der Biodiversitätsverlust, neu auftretende Infektionskrankheiten, die zu Pandemien führen können, wachsende Ungleichheiten und Kriege. Die daraus folgenden Gesundheitsgefahren können katastrophale Ausmaße annehmen, insbesondere wenn sogenannte Kipppunkte überschritten werden. Beispielsweise kann der Klimawandel schwerwiegende Veränderungen zur Folge haben, auch solche, die bis heute kaum vorhersehbar sind, weil die zugrunde liegenden ökologischen Prozesse zu wenig verstanden sind [1].

Im Zusammenhang mit den anthropogenen Umweltveränderungen und der Destabilisierung des Erdsystems sind neue vielseitige und systemische Gesundheitsansätze wie Eco Health, Geo Health, Conservation Medicine, One Health und Planetary Health entstanden. Dabei haben insbesondere die letzten beiden eine weite Verbreitung und Popularität gefunden. Planetary Health und One Health versuchen mit einer systemischen Herangehensweise die Zusammenhänge im Bereich Umwelt und Gesundheit und darüber hinaus in den Blick zu nehmen und dabei mögliche Lösungen für die umfänglichen Probleme zu finden.

Umweltmedizinische Fragestellungen sind seit Entstehung der Heilkünste von großem menschlichen Interesse und wurden im Laufe der Jahrhunderte durch die jeweiligen gesellschaftlichen und politischen Entwicklungen geprägt. Heutzutage ist es für Ärztinnen und Ärzte in Deutschland möglich, sich für den Bereich Umweltmedizin zu qualifizieren, indem sie den Facharzttitel für „Hygiene und Umweltmedizin“ erwerben oder an einer curricularen Fortbildung teilnehmen, die sich vorrangig an klinisch tätige Ärztinnen und Ärzte anderer Fachrichtungen oder im öffentlichen Gesundheitsdienst richtet [2, 3].

Das Fachgebiet Umweltmedizin befasst sich einerseits mit der klinischen Behandlung von Personen mit umweltassoziierten Krankheiten (Individualebene), andererseits betrachtet es auch die Gesamtbevölkerung im Sinne von Public Health oder Global Health. Die zunehmende Popularität der Ansätze One Health und Planetary Health beeinflusst die umweltmedizinische Denkweise im klinischen Alltag und in der Forschung. Hier ergeben sich neue Sichtweisen und Zielkonflikte, die einer gesellschaftlichen Diskussion bedürfen.

Dieser Diskussionsartikel gibt zunächst einen Überblick über die Entwicklung der Konzepte One Health und Planetary Health. Anschließend werden die Aufgaben der Umweltmedizin im zeitlichen Verlauf dargestellt und die Bedeutung von One Health und Planetary Health bei der Weiterentwicklung dieses Fachbereichs aufgezeigt.

## One Health: Geschichte und Bedeutung

„One Medicine“, das als Vorläufer des Konzepts One Health angesehen werden kann, wird hauptsächlich auf Calvin Schwabe und sein 1964 erschienenes Lehrbuch *Veterinary medicine and human health*, welches die menschliche und tierische Gesundheit und deren Zusammenhänge behandelt, zurückgeführt [4]. Um den Bereich der Ökosysteme/Umwelt erweitert wurde der Begriff im Jahr 2004 im Rahmen der 12 „Manhattan Principles“ der Wildlife Conservation Society. Das darauf aufbauende Konzept One Health legte den Fokus auf die Prävention von neu auftretenden und ansteckenden Krankheiten, insbesondere Zoonosen^1^^,^^2^. Der Mensch mit seiner Gesundheit wird als Teil des Tierreichs betrachtet, das wiederum Teil einer gemeinsamen Umwelt ist [5]. Über die letzten Jahre, insbesondere im Kontext der Antibiotikaresistenzen und des Arzneimitteleinsatzes in der Massentierhaltung, wurde das Konzept auch außerhalb von Fachkreisen bekannt. Seit der COVID-19-Pandemie nimmt die Bedeutung von One Health als wichtiger Ansatz im Gesundheitsschutz und speziell Pandemieschutz weiter zu [6].

In den letzten Jahren fand eine Erweiterung des One-Health-Ansatzes statt, da ein zu starker Fokus auf Veterinärmedizin und die Vernachlässigung der Umwelt zunehmend kritisiert wurden. Lagen die Schwerpunkte von One Health in den vergangenen zwei Jahrzehnten auf den Themen antimikrobielle Resistenzen, Lebensmittelsicherheit und Zoonosen, griffen die „Berlin Principles“ von Gruetzmacher et al. im Jahr 2019 als Aktualisierung der Manhattan Principles auch Themen wie den Klimawandel und Biodiversität sowie die Vermittlung von Planetary-Health-Ansätzen und ein Bewusstsein für die Existenz einer globalen Gemeinschaft auf [7].

Die Bedeutung und der Schutz der Biodiversität und der Ökosysteme für die Gesundheit und das Wohlergehen der Menschen spielen eine zentrale Rolle in den 10 Kernaussagen der Berlin Principles. Intakte Ökosysteme gelten als Basis für Gesundheit und Wohlergehen des Menschen. Dies ist sowohl in der Prävention als auch im Umgang mit Infektions- und nichtübertragbaren Krankheiten von Bedeutung. Auch sprechen sich die Verfasser*innen der Berlin Principles für eine Stärkung der nationalen und globalen Zusammenarbeit sowie des öffentlichen Sektors aus, damit wissenschaftliche Erkenntnisse in politische Entscheidungen und in die Praxis umgesetzt werden können [7]. Der One-Health-Ansatz erfährt auf diese Weise eine Einbettung in ökonomische und soziopolitische Kontexte, die die grundlegenden Zusammenhänge des Konzepts ausweiten und gesundheitliche Herausforderungen des 21. Jahrhunderts wie Klimawandel, Biodiversitätsverlust, Infektions- und nichtübertragbare Krankheiten einbeziehen. Wurde One Health vor einigen Jahren für eine zu starke Konzentration auf Veterinär- und humanmedizinische Themen kritisiert, sind die Berlin Principles ein Beispiel für die verstärkte Integration des Umweltsektors und das Zusammenrücken der Ansätze Planetary Health und One Health [7].

In Anbetracht der aktuellen bereits genannten komplexen Probleme an den Schnittstellen zwischen Mensch, Tier und Umwelt wurde 2021 das „One Health High-Level Expert Panel“ (OHHLEP) gegründet, ein aus 26 internationalen Mitgliedern bestehendes multidisziplinäres Experten*innengremium. Seine Aufgabe ist es, Partner*innen aus Wissenschaft und Politik in One-Health-Themen zu unterstützen und zu beraten sowie die sektorenübergreifende Zusammenarbeit auch zwischen den Organisationen der „Quadripartite Alliance“ zu stärken, in der die Weltgesundheitsorganisation (WHO), die Weltorganisation für Tiergesundheit (WOAH), die Ernährungs- und Landwirtschaftsorganisation der Vereinten Nationen (FAO) und das Umweltprogramm der Vereinten Nationen (UNEP) im Rahmen von One Health zusammenarbeiten.^3^ Diese hat 2022 den gemeinsamen „One Health Joint Plan of Action“^4^ veröffentlicht, der die Zusammenarbeit in Bezug auf Gesundheitsgefahren im Anthropozän fördern und strukturieren soll.

**Figure 1:**
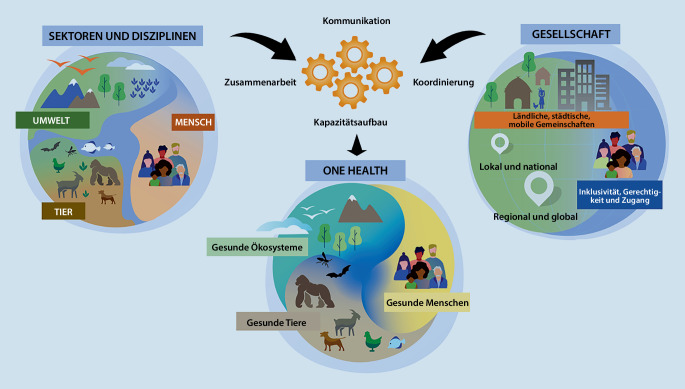

Um ein gemeinsames Verständnis von One Health zu schaffen, erarbeitete das OHHLEP eine Definition des Konzepts, die auf breite internationale Zustimmung stieß. Die zugehörige Grafik (Abb. 1) zeigt, wie die Umsetzung theoretischer Inhalte von One Health in die Praxis gelingen kann. Hierfür sind die vier „K“: Kommunikation, Koordination, Kapazitätsaufbau und Kollaboration (als englische Begriffe: „communication“, „coordination“, „capacity“ und „collaboration“) essenziell, um Gesundheit und Wohlergehen für Menschen, Tiere und Ökosysteme zu ermöglichen [8, 9].

## Planetary Health: Geschichte und Bedeutung

Der Ansatz Planetary Health betrachtet – ähnlich wie One Health – die menschliche Gesundheit als integrativen Bestandteil intakter Ökosysteme der Erde. Das Konzept wurde erstmals 2014 von Richard Horton und Kolleg*innen in einem Artikel des *Lancet* vorgestellt und 2015 im Bericht von Whitmee et al. definiert [10].

Der Bericht beschreibt die anthropogenen Auswirkungen auf die natürlichen Systeme des Planeten und stellt zivilisatorische Erfolge der vergangenen Jahrzehnte (z. B. Steigerung der Lebenserwartung und Verringerung von Armut) den negativen globalen Entwicklungen (z. B. steigender Energie- und Wasserverbrauch, Verlust von Biodiversität und des tropischen Regenwalds, zunehmende Erderwärmung) gegenüber. Die meisten dieser Trends beginnen mit der Industrialisierung gegen Ende des 19. Jahrhunderts und steigern sich besonders ab den 1950er-Jahren [10]. Die starke Zunahme menschlicher Aktivitäten auf der Erde ab der zweiten Hälfte des 20. Jahrhunderts wird als „große Beschleunigung“ (Great Acceleration) bezeichnet [11].

Whitmee et al. zufolge ist ein integrierender Umgang mit den politischen, ökonomischen und sozialen Systemen der Erde erforderlich, die den Zustand natürlicher Systeme wesentlich beeinflussen und somit auch menschliche Gesundheit, Wohlergehen und Gerechtigkeit ermöglichen. Gegenwärtig gefährdet jedoch die Missachtung natürlicher Regenerationskapazitäten von Ökosystemen durch den Menschen die Lebensqualität jetziger und zukünftiger Generationen und droht, bereits erreichte gesundheitliche Fortschritte der vergangenen Jahrzehnte zunichtezumachen [10].

Planetary Health baut auf dem 2009 von Rockström et al. [12] vorgestellten und 2015 von Steffen et al. [14] überarbeiteten Modell der planetaren Belastungsgrenzen auf, welches 9 Systeme definiert, die die Stabilität des gesamten Erdsystems gewährleisten. Hierzu zählen beispielsweise die Bereiche Klimawandel, Integrität der Biosphäre, biogeochemische Flüsse, Versauerung der Ozeane und Landnutzungswandel. Für 7 der 9 Sektoren wurden sogenannte Kipppunkte quantifiziert, deren Überschreitung das Auftreten nicht linearer, relativ abrupt einsetzender und irreversibler Veränderungen für das Erdsystem zur Folge hat, Ökosysteme und Biozönosen^5^ in ihrer Integrität beschädigt und dadurch die sozioökologische Belastbarkeit von Gesellschaften herausfordert. Diese Veränderungen können für Gesellschaften wie auch Individuen katastrophale Ausmaße annehmen. Innerhalb der definierten Belastungsgrenzen liegt der „sichere Handlungsspielraum“ für die menschliche Entwicklung, mit dem das System Erde auch zukünftig in einem Zustand gehalten werden kann, der menschliches Leben unter den besten Bedingungen ermöglicht, ein Zustand, der seit ca. 10.000 Jahren andauert und als Erdzeitalter „Holozän“ bezeichnet wird.

Als Kernsektoren des Modells der planetaren Grenzen gelten die Bereiche Klimawandel und Biosphäre (hierunter wird die funktionale und genetische Vielfalt der Biodiversität verstanden). In diesen Sektoren können bei Überschreitung des sicheren Handlungsspielraums zahlreiche weitere Kipppunkte ausgelöst und auf diese Weise das System Erde in einen Zustand versetzt werden, in dem das Wohlergehen und potenziell auch der Fortbestand der Menschheit gefährdet sind [1, 13]. Laut Steffen et al. hat die Menschheit in den Bereichen Klimawandel, Integrität der Biosphäre mit diversen biogeochemischen Kreisläufen (Austausch von Elementen und Stoffen in unterschiedlichen Biosphärenkomponenten) und Landnutzungswandel den sicheren Rahmen bereits 2015 überschritten [14]. Da die nicht nachhaltige Nutzung von Ressourcen weiter ansteigt, setzt sich diese Entwicklung seitdem weiter fort [15].

Das Modell der planetaren Belastungsgrenzen wird stetig weiterentwickelt. So schlugen Persson et al. im Jahr 2022 Schwellenwerte für den bis dato nicht quantifizierten Bereich der „neuartigen Stoffe“ (hierzu zählen unter anderem nicht natürlich vorkommende Materialien, Chemikalien und Organismen) vor, deren Anwendung ebenfalls auf eine Überschreitung planetarer Kapazitäten in diesem Bereich hinweisen [16]. Im selben Jahr sprachen sich Wang-Erlandsson et al. dafür aus, in den Sektor „globaler Süßwasserverbrauch“, der bis dato ausschließlich das Vorkommen von „blauem Wasser“ (Oberflächen- und Grundwasser) berücksichtigte, zusätzlich „grünes Wasser“ (Niederschläge, Wassergehalt in Böden) zu integrieren. Die Messergebnisse für Bodenfeuchte, welche als Marker für das Maß der Überschreitung dieses Sektors vorgeschlagen wird, deuten darauf hin, dass die Menschheit den sicheren Handlungsspielraum im Bereich des grünen Wassers ebenfalls bereits verlassen hat [17].

Solche Veränderungen der lebenswichtigen Erdsysteme haben gravierende Auswirkungen nicht nur auf die Weiterentwicklung der Menschheit, sondern insbesondere auch auf die Gesundheit und das Wohlbefinden der Menschen. Hitzebedingte Krankheits- und Todesfälle im Zusammenhang mit immer häufiger auftretenden Hitzeextremen infolge des Klimawandels [18] sind nur ein Beispiel für Auswirkungen auf die Gesundheit, die auch in Deutschland bereits spürbar sind.

**Figure 2:**
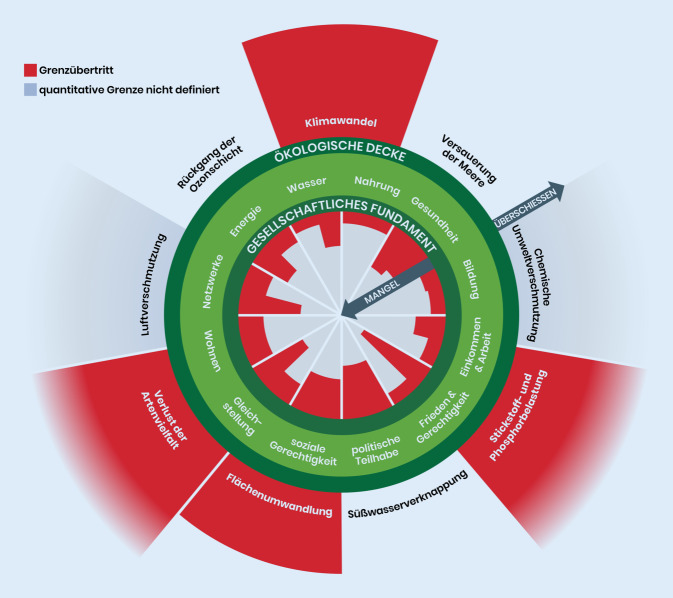

Das 2012 von der Wirtschaftswissenschaftlerin Kate Raworth entwickelte „Donut-Modell“ (Abb. 2) greift den Ansatz der planetaren Grenzen auf und erweitert ihn um soziale Determinanten, beispielsweise den gleichberechtigten Zugang aller Menschen zu Bildung, Wasser, Nahrung und Wohnraum sowie die soziale Chancengleichheit, welche die Gesundheit beeinflussen [19]. Dieses Modell bildet unter anderem die Grundlage des neuen Wirtschaftsmodells der „Ökonomie des Wohlbefindens“^6^^,^^7^. Die Berücksichtigung von planetaren Grenzen und des Wohlbefindens der Menschen in solchen Modellen kann, in die Praxis umgesetzt, positive Wirkungen auf unsere Gesundheitssysteme entfalten. In diesem Zusammenhang werden auch mögliche „Dreifachgewinne“ („triple-wins“) beschrieben [20], die sich auf die Bereiche Chancengerechtigkeit in der Gesundheit, soziale Gerechtigkeit und Nachhaltigkeit beziehen. Die Stadt Amsterdam ist eines der ersten Beispiele, bei dem derzeit ein Wirtschaftsmodell in Anlehnung an das „Donut-Modell“ zu einer Kreislaufwirtschaft transformiert wird [21].

Dass Klimaschutz auch Gesundheitsschutz bedeutet, wird insbesondere durch sogenannte Co-Benefits verdeutlicht: Veränderungen, welche im Sinne des Klimaschutzes umgesetzt werden, beinhalten meist einen Zusatznutzen (Co-Benefit) für die Gesundheit und umgekehrt. Beispielsweise ist belegt, dass eine vorwiegend pflanzliche Ernährung nicht nur die natürlichen Ressourcen schützt und klimaschonend ist, sondern u. a. auch das Risiko für Herzkreislauf- und Krebserkrankungen verringert. Ähnliches gilt für umweltschonende Arten der Fortbewegung und andere Lebensstilfaktoren [22].

Im Vergleich zu One Health liegt der Fokus von Planetary Health verstärkt auf den Themen Klimawandel, Biodiversität, Umweltverschmutzung, Umweltgerechtigkeit und gesellschaftliche Transformation, während One Health aufgrund seiner Entstehungsgeschichte schwerpunktmäßig die Schnittstelle zwischen Tier- und Humanmedizin behandelt. Besonders kennzeichnend für Planetary Health sind unter anderem die Aspekte der Transdisziplinarität [23] und der Dringlichkeit der transformativen Maßnahmen [24]. Daher bemühten sich Vertreter*innen des One-Health-Ansatzes in jüngster Zeit um eine verstärkte Integration des Umweltsektors in das Konzept, sodass eine klare Trennung von One Health und Planetary Health inzwischen nicht immer möglich und sinnvoll ist [25]. Beide Ansätze stehen für die integrative Betrachtung von Gesundheitsthemen vor dem Hintergrund weiterer Wissenschaften, wie beispielsweise den Sozial‑, Natur- oder Geisteswissenschaften, wodurch ein umfassenderes Verständnis von Gesundheitsförderung, allgemeiner Prävention, Krankheitsursache, -diagnose und -therapie ermöglicht wird.

Viele Inhalte von Planetary Health überschneiden sich mit denen der Jahrtausende alten Traditionen indigener Bevölkerungsgruppen. Deshalb ist es wichtig zu erwähnen, dass es sich bei Planetary Health um ein westliches, primär wissenschaftlich begründetes Konzept handelt. Im Gegensatz dazu betrachten indigene Völker ihre natürliche Umwelt mehr als Teil ihrer kulturellen Identität, den es zu schützen, zu pflegen und zu respektieren gilt. Obwohl indigene Traditionen nur in ihrem Kontext betrachtet werden können, da sie sich auf den Ort, die Menschen, die dort herrschende Ethik und den Glauben beziehen, können sie die heutigen anthropozentrischen Sichtweisen und Vorstellungen infrage stellen und zu einem nachhaltigeren Umgang mit den Systemen der Erde beitragen [26]. 2014 wurde dem Whanganui-Fluss in Neuseeland, der für die vor Ort lebenden Maori von großer Bedeutung ist, der Status einer juristischen Person verliehen. Beispiele wie dieses zeigen, wie der Wandel menschlicher Vorstellungen über die Natur von reinem Besitz bis zur Anerkennung ihres intrinsischen Wertes möglich ist [27].

## Die Aufgabe und Entwicklung der Umweltmedizin

Obwohl die Umweltmedizin in ihrem heutigen Verständnis ein relativ junges Fachgebiet ist, sind Umwelt- und Gesundheitsthemen seit dem Altertum von menschlichem Interesse [28]. Inhaltlich versteht sich die Umweltmedizin als Wissenschaft, die die Entstehung von Krankheit und die Erhaltung der Gesundheit des Menschen durch Aspekte aus dessen Umwelt erforscht. Der Begriff „Umwelt“ beinhaltet eine gewisse Unschärfe und kann auf vielfältige Weise – beispielsweise als natürliche, soziale, kulturelle oder wirtschaftliche Umwelt – definiert werden. Um dem Fachgebiet einen überschaubaren Rahmen zu geben, wurden in der Umweltmedizin bis heute vorrangig die durch anthropogene Einflüsse entstandenen biologischen, physikalischen und chemischen Faktoren und ihre Wirkungen auf den Menschen behandelt [29].

In Deutschland umfasst dieses wissenschaftlich geprägte Teilgebiet der Medizin einerseits einen primärpräventiven Ansatz im Sinne der Erkennung, Bewertung und Vermeidung von schädlichen Einflüssen aus der Umwelt bezogen auf die Gesamtbevölkerung, das Individuum oder einzelne exponierte Gruppen. Andererseits bezieht sich der klinische Zweig der Umweltmedizin auf die Behandlung und Betreuung von Patient*innen, die bereits veränderte klinische Parameter (Sekundärprävention) oder manifeste umweltbedingte beziehungsweise durch Umweltfaktoren beeinflusste Krankheiten (Tertiärprävention) aufweisen [30]. Der Nachweis einer Kausalität zwischen Umwelteinwirkung und gesundheitlicher Schädigung erweist sich oftmals als schwierig, da die Krankheitsbilder häufig vielfältige Symptome aufweisen und durch weitere Faktoren – beispielsweise den psychosozialen Kontext, die genetische Prädisposition oder die individuelle Empfindlichkeit – beeinflusst werden [31].

Im Laufe des 20. Jahrhunderts nahmen umweltmedizinische Fragestellungen eine zunehmend globalere Perspektive ein, die anthropogene Einflüsse auf die Umwelt stärker berücksichtigte. Veröffentlichungen, wie *Silent spring* von Rachel Carson (1962), *Limits to growth* des Club of Rome (1972), die Ottawa Charta (1986) sowie Umweltkatastrophen wie das Seveso-Unglück 1976 oder Tschernobyl 1986 sorgten für ein bis dato kaum existierendes kollektives Umweltbewusstsein. Hierbei wurden insbesondere die komplexen Verstrickungen zwischen Menschen und Umwelt hervorgehoben, welche „die Grundlage für einen sozioökologischen Weg zur Gesundheit“ [32] darstellen. Diese Entwicklungen beeinflussten die Umweltbewegung der 1970er- und 1980er-Jahre maßgeblich und hatten in Deutschland zum Beispiel die Gründung der Partei „Die Grünen“ oder des Umweltministeriums zur Folge. Zudem befeuerten diese Entwicklungen die Diskussionen zum Thema „Umwelt und Gesundheit in den Industrienationen“ [33, 34]. In Deutschland spiegelte sich die Anerkennung der engen Verbindung zwischen Ökologie und Medizin bereits in der Approbationsordnung von 1972 wider, in der die Gebiete Sozial‑, Arbeits‑, Rechtsmedizin, Hygiene, Informationsverarbeitung und medizinische Statistik unter dem Begriff „ökologische Fächer“ gebündelt wurden [30].

Im internationalen Vergleich erweist sich eine länderübergreifende Betrachtung des Feldes Umweltmedizin als schwierig, da sich die Einflüsse auf die Entwicklung umweltmedizinischer Strömungen zwar ähneln, die Institutionalisierung und der Umgang mit umweltmedizinischen Inhalten aber teilweise sehr verschieden sind. So existieren im englischsprachigen Raum sowohl der Begriff „environmental health“ als auch „environmental medicine“; Ersterer begreift sich eher als übergreifender Bereich mit Fokus auf Public Health, während Letzterer sich auf den klinischen Bereich der Umweltmedizin bezieht [35]. Die Abgrenzung ist aber unscharf, teilweise werden beide Begriffe synonym verwendet.^8^ Auch die deutsche Universitäts- und Forschungslandschaft wird mittlerweile von internationalen umweltmedizinischen Fachrichtungen beeinflusst, wie die Einrichtung eines Forschungsschwerpunktes der medizinischen Fakultät der Universität Augsburg unter dem Namen des verwandten Fachgebiets „Environmental Health Sciences“ aus den USA zeigt.^9^

## Die Bedeutung von One Health und Planetary Health für die Umweltmedizin

Der Blick auf umweltmedizinische Fragestellungen ist oft ein Spiegel der jeweiligen zeitgeschichtlichen Geschehnisse und aktuellen gesellschaftlichen Aufgaben. Besonders durch die Entwicklungen des 20. Jahrhunderts wird der Fokus der Umweltmedizin auf anthropogene Umwelteinflüsse und ihre gesundheitlichen Auswirkungen gerichtet. Aktuelle Beispiele wie der Klimawandel, der Biodiversitätsverlust und Pandemien sowie deren Folgen zeigen, dass bereits heute irreversible Veränderungen unserer Umwelt eingetreten sind, die sich auf die Gesundheit der Weltbevölkerung auswirken. Die Folgen des Umgangs der Menschen mit den natürlichen Systemen der Erde sind vielfältig, miteinander verzahnt und betreffen infolge der Globalisierung und des schieren Ausmaßes menschlichen Ressourcenverbrauchs und Konsums die ganze Welt. Hier spielt der Aspekt der Umweltgerechtigkeit eine zentrale Rolle: Reiche Nationen im globalen Norden verursachen deutlich höhere CO_2_-Emissionen und sind hauptverantwortlich für die Überschreitung planetarer Grenzen, deren Auswirkungen ärmere Länder im globalen Süden am stärksten zu spüren bekommen [36]. Gesundheitliche Belastungen durch anthropogene Umwelteinflüsse sind seit Beginn des industriellen Zeitalters stetig fortgeschritten und heutzutage überall möglich, die Trennung zwischen natürlicher, unberührter Natur und menschlicher Besiedelung ist zunehmend fraglich [28]. Umweltverschmutzungen können in den entlegensten Gebieten der Erde nachgewiesen werden, die Folgen des Klimawandels sind mess- und sichtbar [37, 38].

Während One Health zwar eine integrative Sichtweise von Mensch, Tier und Umwelt anstrebt, findet bei Planetary Health eine Ausweitung des Gesundheitsbegriffs auf den ganzen Planeten statt. Dies bedeutet nicht nur die enge Verbindung von Systemen weltweit, sondern auch die Einbeziehung künftiger Generationen, wie die Ursprungsdefinition im Artikel von Whitmee et al. bereits andeutet. Wurde bis vor einigen Jahren in der klinischen Umweltmedizin hauptsächlich die lineare kausale und überwiegend lokale Wirkungskette zwischen Umwelteinfluss und Individuum betrachtet, muss im Sinne von Planetary Health und One Health eine systemische Herangehensweise bezüglich Krankheitsätiologie, -diagnose und -therapie gewählt werden.

So hat nicht nur ein Umweltfaktor multiple Wirkungen auf die menschliche Gesundheit auf individueller und Bevölkerungsebene, auch können Diagnose und Therapie im weitesten Sinne globale Auswirkungen haben. Beispielsweise haben Krankenhäuser und Praxen derzeit einen hohen Verbrauch an Einwegprodukten, der häufig damit begründet wird, dass Hygienevorgaben besser eingehalten werden können. Auch das Medikament Diclofenac, das in verschiedenen Darreichungsformen (auch für die Lokalanwendung) häufig verschrieben wird, führt zu einer Belastung der Gewässer mit Folgen für Wasserorganismen [39].

Obwohl die Sinnhaftigkeit der Anwendung dieser Produkte in vielen Fällen begründet ist, müssen in einer Gesamtbetrachtung die Auswirkungen auf die Gesundheit aller Menschen in der Gegenwart und in der Zukunft mit beachtet werden. So können zum Beispiel hygienische Maßnahmen, die den gesundheitlichen Schutz eines Einzelnen bedeuten, die Gesundheit jetziger und künftiger Generationen beeinträchtigen, wenn sie nicht nachhaltig sind und die Umwelt schädigen.

Die Einbeziehung globaler Umwelt- und Gesundheitseffekte in bisher vorwiegend auf lokaler Ebene betrachtete Sachverhalte zeigt, dass im Sinne von Planetary Health und One Health eine umweltmedizinische Betrachtung immer auf zwei Ebenen erfolgen muss – zuerst wird die Ebene des individuellen Falls betrachtet und im zweiten Schritt die regionalen, nationalen und globalen Auswirkungen, die beispielsweise durch Ressourcenverbrauch oder Schädigung der Umwelt ausgelöst werden. Vor diesem Hintergrund kann die Schwierigkeit entstehen, dass in konkreten Fällen der Gesundheitsschutz verschiedener Personengruppen zu unterschiedlichen Zeitpunkten (heute und in der Zukunft) gegeneinander aufgewogen und priorisiert werden oder gegebenenfalls ein Kompromiss gefunden werden muss. Unter Berücksichtigung der Konzepte One Health und Planetary Health braucht es eine Diskussion in nahezu allen medizinischen Bereichen (hier liefert die Zusammenstellung von Traidl-Hoffmann et al. einen guten Überblick über viele betroffene Fachdisziplinen [40]). Oftmals wird dies die Abwägung einer medizinischen Evidenz (z. B. der belegbare Vorteil von Einwegprodukten) gegenüber dem erhöhten Ressourcenverbrauch oder umweltschädlichen Einflüssen, welche ihrerseits gesundheitsschädlich wirken können, bedeuten.

In diesem Zusammenhang ist ebenfalls eine gesellschaftliche Diskussion erforderlich. Letztere bedarf einer Berücksichtigung gesundheitlicher Fragestellungen in allen Politikfeldern, im Sinne eines „Health-in-all-Policies“-Ansatzes. Interventionen umwelt- und gesundheitspolitischer Art können gemäß dem Konzept der „sozialen Kipppunkte“ z. B. über Multiplikatoreneffekte und die Beseitigung von Fehlanreizen wirkungsvolle Transformationsprozesse innerhalb der Gesellschaften anstoßen.

Da der Umweltmedizin die Aufgabe zukommt, umweltbedingte Gesundheitseffekte zu bewerten und Lösungsvorschläge auf individueller wie auf Bevölkerungsebene zu unterbreiten, ist es wahrscheinlich, dass in diesem Bereich durch die beiden neuen Konzepte ein Paradigmenwechsel in der Herangehensweise an Bewertungsfragen eintreten wird. Sowohl in der individuellen als auch in der bevölkerungsbezogenen Umweltmedizin (dem Bereich der sog. Environmental Public Health) werden in Zukunft Fragen der Nachhaltigkeit in Beratungs- und Bewertungsprozessen eine wesentlich größere Rolle spielen.

## Footnotes

1 Zoonosen sind Infektionskrankheiten, die gleichermaßen bei Tieren u. Menschen vorkommen und sowohl vom Tier auf den Menschen als auch vom Menschen auf Tiere übertragen werden können.

2 http://www.oneworldonehealth.org/sept2004/owoh_sept04.html, Zugegriffen: 17.01.2023.

3 https://www.who.int/news/item/11-06-2021-26-international-experts-to-kickstart-the-joint-fao-oie-unep-who-one-health-high-level-expert-panel-(ohhlep), Zugegriffen: 01.12.2022.

4 https://www.unep.org/resources/publication/one-health-joint-plan-action-2022-2026, Zugegriffen 13.01.2023.

5 Als Biozönosen werden Lebensgemeinschaften von Organismen unterschiedlicher Arten in einem relativ kleinen abgeschlossenen Bereich (Biotop) bezeichnet.

6 https://www.consilium.europa.eu/de/infographics/economy-wellbeing/, Zugegriffen: 01.12.2022.

7 https://eurohealthnet.eu/de/publication/an-economy-of-wellbeing-for-health-equity/, Zugegriffen: 01.12.2022.

8 https://www.eea.europa.eu/help/glossary/eea-glossary/environmental-medicine, Zugegriffen: 01.12.2022.

9 https://www.uni-augsburg.de/de/forschung/einrichtungen/institute/wzu/forschung/environmental-health-sciences/, Zugegriffen: 01.12.2022.
